# Identification of Biomarkers Based on Starvation Response-Related Genes for Assessing the Immune Profile and Prognosis in Lung Adenocarcinoma

**DOI:** 10.1155/ijog/9950674

**Published:** 2025-07-11

**Authors:** Xixian Lou, Hui Xia, Zongxiao Shangguan, Lianmin Bao, Heping Lin

**Affiliations:** ^1^Department of Respiratory Medicine, The Third Affiliated Hospital of Wenzhou Medical University, Rui'an, China; ^2^Ultrasound Imaging Department, The Third Affiliated Hospital of Wenzhou Medical University, Rui'an, China

**Keywords:** autophagy, glycolysis, long-stranded noncoding RNA, lung adenocarcinoma, starvation

## Abstract

**Background:** Lung adenocarcinoma (LUAD) exhibits a high recurrence rate and an unfavorable prognosis. The role of the starvation-induced tumor microenvironment (TME), which is closely linked to metabolism, remains poorly understood in LUAD.

**Methods:** LUAD patient samples were collected from public databases, and starvation response-related genes (SRRGs) were acquired from the MSigDB database. As starvation response may enhance autophagy in cancer cells, the Human Autophagy Database (HADb) was accessed to collect autophagy-related genes (ARGs). Next, the association between the expressions of ARGs and SRRGs was analyzed applying Pearson's algorithm. The SRRG score was calculated by GSVA package for each sample, and WGCNA package was utilized to screen SRRG-related module genes. Differentially expressed lncRNAs between LUAD and control samples were screened by the limma package. Subsequently, the lncRNAs associated with SRRG-related module genes were intersected with differentially expressed lncRNAs to obtain key SRRG-correlated lncRNAs. The number of key lncRNAs in the risk model was optimized by performing univariate and multivariate Cox regression analyses. Next, immune profiling between different LUAD risk groups was conducted using single-sample GSEA (ssGSEA), MCP-counter, and ESTIMATE algorithms. The Mutect2 software and clusterProfiler R package were employed to analyze the mutation profiles and pathway enrichment of patients in different risk groups, respectively. In addition, the expressions of key lncRNAs in LUAD cells were verified by qRT-PCR, and the migratory and invasive capabilities of the cells were measured by wound healing and transwell assays.

**Results:** We identified 162 potential SRRGs linked to autophagy, glycolysis, and starvation responses. In addition, 102 candidate SRRG-related lncRNAs were selected from SRRG-related module genes. Three key SRRG-related lncRNAs (AC023421.1, AL034397.3, and LINC01537) were screened for developing an accurate risk model. Notably, the high-risk group showed a significantly higher mutation rate and oncogenic pathway scores and markedly worse immune infiltration and overall survival (OS). In vitro experiments revealed that LINC01537 was highly expressed in A549 cells, and that after knockdown of LINC01537, the migration and invasion of LUAD cells were suppressed.

**Conclusion:** This study identified three key lncRNAs related to starvation response and created a risk model that can accurately assess the prognosis and immune characteristics in LUAD, offering novel biomarkers and a theoretical basis for the precision immunotherapy and targeted intervention in LUAD.

## 1. Introduction

Lung adenocarcinoma (LUAD), a frequently detected lung cancer subtype, shows the highest mortality in the world [1, 2]. Though LUAD treatment has been advanced, the 5-year overall survival (OS) of patients suffering from LUAD remains lower than 20% [3]. For advanced or metastatic LUAD, early surgery and systemic therapy can relieve the symptoms and modestly prolong survival [4, 5]. However, chemoresistance and metabolic changes typically restrict the antitumor efficacy of the treatment, leading to tumor recurrence and a poor prognosis [6, 7]. Thus, discovering innovative and effective prognostic biomarkers is essential for accurately identifying high-risk LUAD patients who are likely to have a poor prognosis, contributing to the development of novel therapeutic strategies for LUAD patients [8, 9].

The rapid proliferation of tumors often outpaces its nutritional reserves, leading to a hostile tumor microenvironment (TME) characterized by a lack of glucose and deficiency of amino acids [10]. Hence, the capability to adapt to such challenging TME is essential for cancer cell survival. A study showed that cancers, such as breast cancer, are capable of optimizing resource utilization to endure a nutrient-depleted TME [11, 12]. Under starvation circumstances, cancer cells adaptively upregulate key metabolic enzymes of the glycolytic pathway to ensure energy supply and increase glucose consumption [13]. In particular, cancer cells that undergo extreme energy deprivation and starvation will promote autophagy and convert their metabolic processes to “eat” and “digest” their cytoplasmic components, allowing the cells to survive in unfavorable environments [14–16]. As a diverse category of transcripts, lncRNAs are increasingly recognized as important regulators involved in gene expressions and a variety of pathological and physiological processes [17]. lncRNAs can enhance energy metabolism and facilitate cancer progression by modifying key metabolism-related proteins through posttranslational processes, such as ubiquitination, phosphorylation, and acetylation [18–20]. At present, research on the molecular mechanisms that facilitate the progression of LUAD within a starved TME remains limited. Hence, this study was designed to identify crucial starvation response-related genes (SRRGs) as potential biomarkers for the survival evaluation in LUAD, hoping to offer important insights into energy stress-related signaling pathways in LUAD.

This study selected candidate SRRGs from public databases related to starvation, autophagy, and glycolysis. Weighted gene coexpression network analysis (WGCNA) was employed to classify gene modules showing the strongest correlation with candidate SRRG scores. The resulting gene expression profiles were then intersected with differentially expressed lncRNAs between LUAD and control samples to screen for key prognostic genes. Further, we constructed a Riskscore system to stratify LUAD patients into high- and low-risk groups. The correlation between the Riskscore and immune characteristics, mutation rate, and oncogenic pathway scores of LUAD patients was evaluated, and the functional enrichment of the two risk groups was conducted. Overall, we established an accurate prognostic prediction model integrating starvation, autophagy, and glycolysis features, hoping to improve the assessment of immune characteristics and prognostic prediction for LUAD patients.

## 2. Methodology

### 2.1. Data Collection and Processing

TCGA database (https://portal.gdc.cancer.gov/) was accessed to obtain the clinical data and gene expression profiles of LUAD, and RNA-Seq data were processed by transcripts per million (TPM) standardization. During the processing of the TCGA-LUAD data, all patients included had a survival time of > 0 day while eliminating those with missing survival time or status. Ultimately, 500 LUAD samples and 59 control samples were retained from the TCGA database. Additionally, GEO database (https://www.ncbi.nlm.nih.gov/geo/) was accessed to download the dataset GSE31210. After probe-to-gene symbol conversion using annotation files and excluding samples without OS or clinical follow-up data, 226 tumor samples were retained for further analysis.

Next, gene set enrichment analysis (GSEA) was performed by utilizing the SRRGs from the “GOBP RESPONSE TO STARVATION” gene set and another 62 glycolysis-related genes (GRGs) collected from the MSigDB database (https://www.gsea-msigdb.org/gsea/msigdb/index.jsp). Glycolysis-related SRRGs were filtered using Pearson correlation analysis (*r* > 0.3, *p* < 0.05). Next, the HADb (http://www.autophagy.lu) was accessed to retrieve 232 autophagy-related genes (ARGs). The association between the expressions of ARGs and SRRGs was examined using Pearson correlation coefficients. Autophagy-correlated SRRGs were screened under *r* > 0.3 and *p* < 0.05. Lastly, SRRGs linked to autophagy and glycolysis were intersected to take common genes, and those near the intersection were considered potential SRRGs.

### 2.2. WGCNA for Key Gene Identification

With candidate SRRGs as background gene sets, the scores of candidate SRRGs for each TCGA cohort sample were computed independently by ssGSEA [21]. Gene modules linked to the score were classified by WGCNA. To effectively detect close correlations between modules, the pickSoftThreshold function of “WGCNA” R package was used to establish the optimal soft threshold power (*β* = 4) [22]. Then, gene modules were identified by hierarchical cluster analysis under the parameter of minModuleSize = 60. Lastly, based on the first principal component of the module expression, various module–trait genes were obtained using the “heatmap” R package [23]. Module–trait correlations were assessed based on the association between the module genes and candidate SRRG scores. The component genes in the modules showing the strongest trait associations were analyzed.

### 2.3. Analysis of lncRNAs Associated With SRRGs in LUAD

Using the “limma” R package [24], differently expressed lncRNAs across LUAD and control samples in the TCGA cohort were identified. To screen lncRNAs with significant expression, gene expression profiling data were processed through background adjustment and quantile normalization, with |log2(FC)| > log2(1.2) and padj < 0.05 as significance thresholds. Differentially expressed lncRNAs with padj < 0.05 were considered statistically significant. SRRG-associated lncRNAs were obtained by intersecting module gene-associated lncRNAs and differentially expressed lncRNAs (Cor > 0.5, *p* < 0.05).

### 2.4. Establishment of a Risk Model and Verification

Prognostically significant genes (*p* < 0.05) in the TCGA-LUAD training set were selected by subjecting SRRG-related lncRNAs to univariate Cox proportional risk regression using the “survival” R package [25]. Independent prognostic factors in the TCGA-LUAD training set were identified by multivariate Cox regression analysis. Riskscore was formulated as Riskscore = *Σβ*i × Expi for each patient. Expi is the expression of each gene, *i* denotes gene expression, and *β* is the Cox regression coefficient of a gene. After z-score normalization, the TCGA-LUAD training set was classified into low-risk and high-risk groups by the optimal cutoff of the Riskscore. Survival analysis for the two risk groups was performed using the “survminer” R package [26]. Prognosis analysis was conducted employing Kaplan–Meier (KM) curves and the log-rank test. Additionally, the model robustness was tested according to ROC curves plotted by the “timeROC” R package [27]. Area under the curve (AUC) for OS prediction during 1, 2, 3, 4, and 5 year(s) of period was computed for the TCGA-LUAD training set. GSE31210 dataset was similarly processed for the verification.

### 2.5. TME Analysis of LUAD

We next used the ssGSEA function of the “GSVA” R package [28] to compute the scores of 28 types of tumor-infiltrating immune cells in the TCGA-LUAD cohort. The “MCP-counter” R package [29] was applied to examine the relationship between the Riskscore and the scores of the 10 immune cells in the TCGA-LUAD dataset [26]. The “estimate” R package [30] was employed for the correlation analysis between Riskscore and LUAD immune function in the TCGA-LUAD dataset.

### 2.6. Analysis of Cytolytic Activity (CYT) Scores, IFN-*γ* Scores, and Mutational Landscapes in Low- and High-Risk Groups

Perforin 1 (*PRF1*) is a pore-forming enzyme that facilitates the entry of granzymes into target cells, while granzyme A (*GZMA*) is an enzyme that resembles trypsin and causes caspase-independent apoptosis. Immune cells in the TME function crucially in controlling tumor biology and overexpressed *PRF1* and *GZMA*, cytotoxic T lymphocytes, and NK cells can destroy tumor cells [31]. CYT [32], which is calculated as the geometric mean of *PRF1* and *GZMA* [33], demonstrates a close correlation with a better patient survival. Here, the depth of coverage estimate was determined by dividing the total raw read counts for each gene by its maximum transcript length. Following a 0.01 offset to eliminate zero counts from the computation, coverage estimates were scaled to a total depth of 1e^6^ in each sample and obtained as TPM. CYT high and low cohorts of the TCGA-LUAD patients were classified and compared, with each cohort having equal histology-stage combinations. FDR-adjusted *p* value was used for CYT comparisons between samples. Each sample was assigned a score calculated by ssGSEA based on the Th1/IFN-*γ* gene signature collected from an earlier work [34]. Gene mutations in each TCGA-LUAD sample were also calculated as they were closely involved in LUAD development [35]. Mutect2 software [36] was utilized to process the mutation dataset of TCGA-LUAD samples and to map the gene mutations in the two risk groups.

### 2.7. Enrichment Analysis of High- and Low-Risk LUAD Groups

Using the “limma” R package, the differentially expressed genes (DEGs, |Fold Change| > 1.5 and FDR < 0.05) between the risk groups were identified. The “clusterProfiler” R package [37] was used to perform Gene Ontology (GO) and Kyoto Encyclopedia of Genes and Genomes (KEGG) on the DEGs. Bubble plots were plotted for the top 10 functions enriched in the three terms of GO analysis [38] and the top 8 pathways in the KEGG analysis results. Additionally, the enrichment score for 10 tumor-associated pathways [38] was computed by ssGSEA, followed by using Kruskal for the comparative test [39].

### 2.8. Immune Characterization of LUAD

TIDE is a computational tool [40] that indicates low responsiveness to immune checkpoint inhibition (ICI) therapy by a high TIDE score. Here, we compared the dysfunction and exclusion scores between the two subsets. Then, Pearson's correlation showed that mRNAs and lncRNAs had a positive correlation (Cor > 0.25 and *p* < 0.05) in TCGA. The “WebGestaltR” R package [41] was employed to conduct KEGG analysis on the mRNAs positively linked to the lncRNAs.

### 2.9. Cell Culture and siRNA Transfection

From the Institute of Biochemistry and Cell Biology, Chinese Academy of Sciences, human lung cancer cells (A549) and human lung normal epithelial cells (BEAS-2B) were purchased for the current experiments. RPMI 1640 media (Gibco, Grand Island, NY, United States) with 100 mg/mL streptomycin (Invitrogen, Carlsbad, CA, United States), 10% fetal bovine serum (10% FBS), and 100 U/mL penicillin were employed for culturing all the cells with 5% CO_2_ at 37°C in humidified air.

The siRNAs of LINC01537 were designed and synthesized by IGEbio (Guangzhou, China) to knock down LINC01537. Following the instructions, the LUAD cells were transfected with siRNAs utilizing Lipofectamine 3000 Transfection Reagent (Invitrogen, CA, United States). After 24 h, the transfection efficiency was tested by qRT-PCR. All siRNA sequences were as follows: si-LINC01537#1 (sense: 5⁣′-GCUGGAGGCCUCCAAGAAATT-3⁣′; antisense: 5⁣′-UUUCUUGGAGGCCUCCAGCTT-3⁣′) and si-LINC01537#2 (sense: 5⁣′-GCAUCUACUCACCAUCCUUTT-3⁣′; antisense: 5⁣′-AAGGAUGGUGAGUAGAUGCTT-3⁣′).

### 2.10. RNA Extraction and qRT-PCR

Following the instructions, total RNA from A549 and BEAS-2B cells was isolated using a RNA Extraction Kit (TRIzol, Invitrogen, United States). The HiScript II kit (Vazyme, Nanjing, China) was employed to synthesize cDNA templates after determining the purity and concentration of the total RNA. The KAPA SYBR Fast kit (Sigma-Aldrich, St. Louis, MO, United States) and specific primers were applied to carry out qRT-PCR. With GAPDH as an internal control, the data were computed by the method of 2^-ΔΔCT^. Primer sequences for the genes utilized were shown in Table 1.

### 2.11. Wound Healing Assay

The effect of LINC01537 expression on the migration and invasion of A549 cells was analyzed by performing scratch and transwell assays. To measure cell migration with the wound healing assay, 2 mL of cell suspension of transfected cells was inoculated into 6-well plates (5 × 10^5^/mL) and incubated with 5% CO_2_ at 37°C in an incubator. A 10-*μ*L plastic pipette tip was employed to uniformly scratch the monolayer when the cell concentration reached approximately 80%. The monolayers were then washed with PBS, followed by incubation in FBS-free medium. Wound distances of the migrating cell sheet were photographed at 0 and 48 h. All the experiments were performed in three sets.

### 2.12. Transwell Assay

Cell invasion was measured using transwell chambers (Corning, Inc., Corning, NY, United States). After 36 h of transfection, the matrix gel-coated upper chamber (BD Biosciences, Franklin Lakes, NJ, United States) contained 5 × 10^5^ cells in 200 *μ*L of nonserum medium, while 700 *μ*L of medium with 10% FBS was supplemented in the lower chamber. After incubation for 48 h, 0.1% crystal violet solution was applied to stain the cells in the lower chamber, followed by using 4% paraformaldehyde for fixation. Finally, the cells were photographed from six randomly chosen fields of view under a microscope and quantified.

### 2.13. Statistical Tests

Prism 8 (GraphPad Software, San Diego, CA, United States) and R software (Version 3.6.0, R Foundation, Vienna, Austria) were used in all statistical analyses. The Wilcoxon rank-sum test determined differences between the two sets of continuous variables. Differences between the KM survival curves were evaluated. The log-rank test was employed to examine survival time differences among each group, and the correlations were computed using the Spearman method. One-way analysis of variance or unpaired *t*-test was applied to compare the experimental data. A *p* < 0.05 denoted statistical significance.

## 3. Results

### 3.1. Candidate SRRGs and Construction of Coexpression Networks

**Figure 1:**
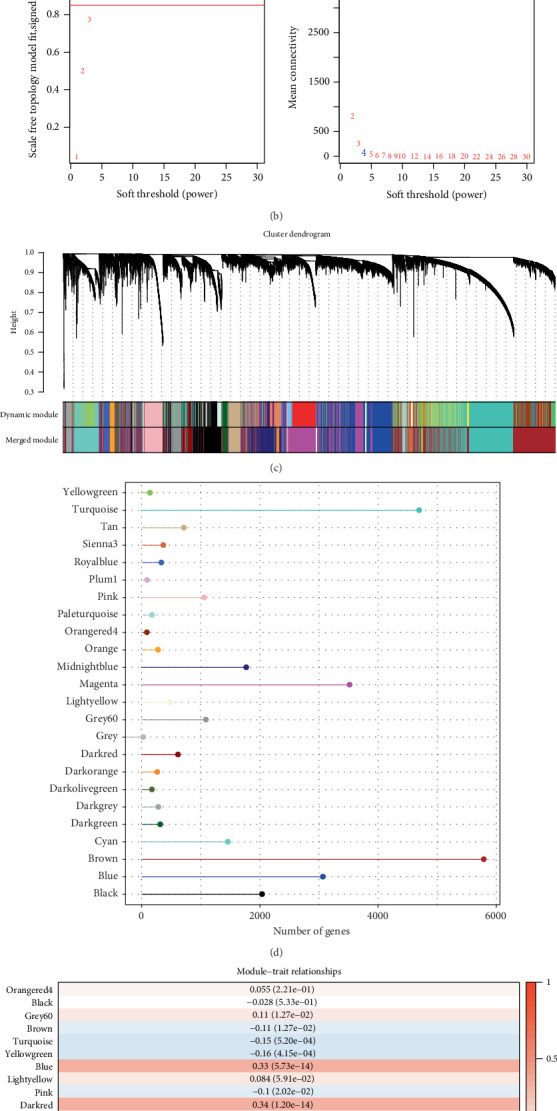
Identification of candidate hub genes based on WGCNA. (a) Intersection of autophagy-related SRRGs and glycolysis-related SRRGs. (b) Various soft threshold powers (*β*) were subjected to scale-free fit index analysis, and various soft threshold powers were subjected to average connectivity analysis. (c) Dissimilarity metric (1-TOM) clustering in gene dendrogram. (d) Gene number in each module. (e) Correlation of module eigenvectors with features for each module.

Genes linked to autophagy and starvation response were first analyzed. A sum of 186 autophagy-related SRRGs was obtained between 217 SRRGs from MSigDB and 232 ARGs from the HADb database. In MSigDB, there were 62 GRGs related to 217 SRRGs. A total of 171 glycolysis-associated SRRGs were identified based on the coexpression correlation between the expressions of the 217 SRRGs and GRGs. A total of 162 genes associated with both starvation, autophagy, and glycolysis were therefore considered the candidate SRRGs for further research (Figure 1a). WGCNA analysis adjusted the soft threshold to 4 in the TCGA cohort to ensure a scale-free network (Figure 1b). We created a gene hierarchy clustering dendrogram by analyzing gene correlations and found 24 unique gene modules with coexpression patterns (Figure 1c). As shown in Figure 1d, among the 24 modules, the number of genes in the brown and turquoise modules was relatively high, followed by the magenta module. Modules closely linked to the candidate SRRG were classified based on the candidate SRRG scores calculated by the R package “GSVA” for each TCGA cohort sample, and the correlation between each module and candidate SRRG scores was analyzed. Our analysis demonstrated that the dark red module was closely positively related to the candidate SRRG scores (Cor = 0.34, *p* = 1.20e − 14, Figure 1e).

### 3.2. Identification of lncRNAs Associated With Candidate SRRGs in LUAD

**Figure 2:**
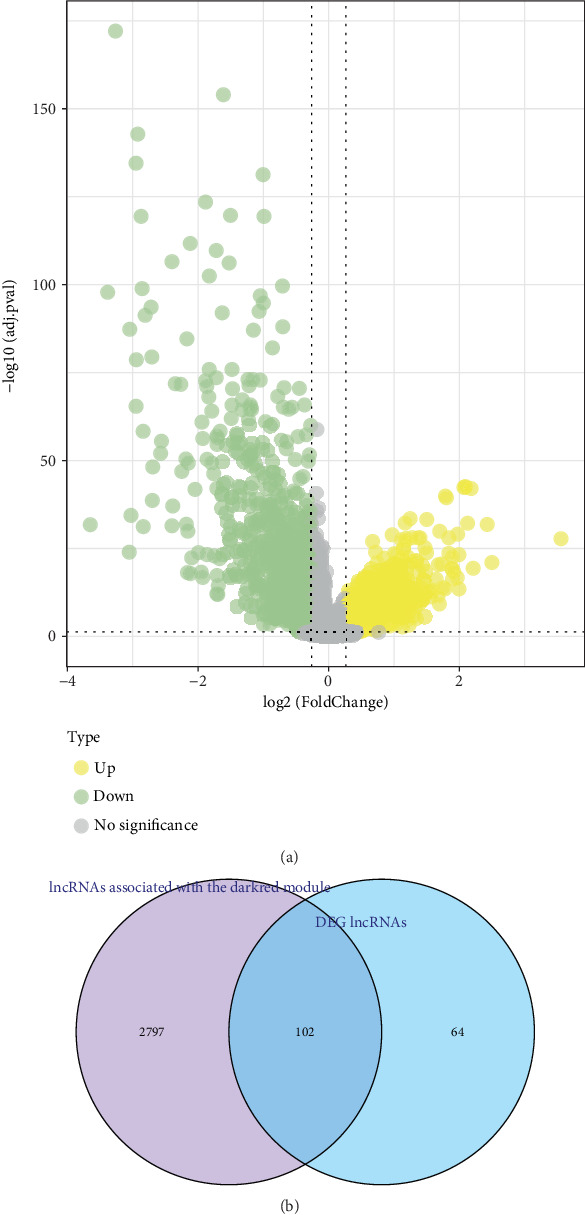
lncRNAs linked to the candidate SRRGs in the TCGA cohort. (a) Differential lncRNA volcano plot between TCGA cohort control samples and LUAD samples. (b) The intersection of differential lncRNAs and dark red module gene-associated lncRNAs.

Differentially expressed lncRNAs in LUAD and control samples of the TCGA cohort were visualized in a volcano plot (Figure 2). Finally, 2899 differentially expressed lncRNAs, comprising 1774 upregulated lncRNAs and 1125 downregulated lncRNAs, were screened. The lncRNAs linked to the genes in the dark red module were then computed, with Cor > 0.5 and *p* < 0.05 as the filtering criteria. Subsequently, 102 lncRNAs linked to potential SRRGs were obtained by intersecting the differential and dark red module gene-related lncRNAs (Figure 2).

### 3.3. Establishment of a Clinical Prognostic Model and Validation

**Figure 3:**
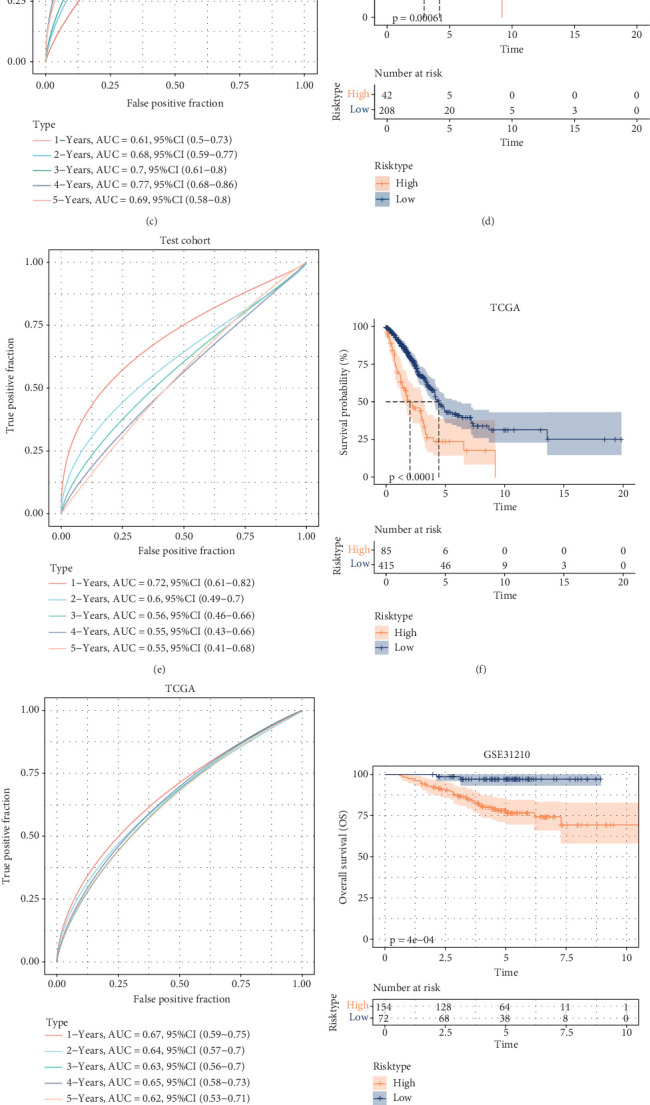
Establishment of a Riskscore model and validation. (a) Coefficient distribution of each prognosis gene. (b) Kaplan–Meier survival curves for the TCGA-LUAD training data cohort. (c) ROC curves of the Riskscore in the TCGA-LUAD training data cohort. (d) Kaplan–Meier survival curves for the TCGA-LUAD validation cohort. (e) ROC curve for the Riskscore in the TCGA-LUAD validation data cohort. (f) Kaplan–Meier survival curves for the TCGA cohort. (g) The Riskscore in the TCGA cohort assessed by ROC curve. (h) Kaplan–Meier survival curves for the Riskscore in the GSE31210 cohort. (i) ROC curve of the Riskscore in the GSE31210 cohort.

The TCGA-LUAD cohort was evenly grouped into training and test sets. After removing redundant confounding genes, genes significantly influencing the prognostic outcomes in the TCGA-LUAD training set were subjected to univariate Cox proportional risk regression using the “survival” R package. Furthermore, multivariate Cox regression analysis determined that three genes (*AC023421.1*, *AL034397.3*, and *LINC01537*) were independently linked to LUAD prognosis (Figure 3a). Subsequently, the prognosis of the patients in the TCGA-LUAD training set was characterized utilizing the formula of the Riskscore: Riskscore = (−0.142∗*AC*023421.1) + (−0.386∗*AL*034397.3) + 0.669∗*LINC*01537. The KM curves showed that in comparison to the high-risk LUAD patients, those with a low risk in the TCGA-LUAD training cohort overall survived longer (*p* < 0.0001, Figure 3b). In the TCGA-LUAD training set, the patients were assigned into low- and high-risk groups by the optimal threshold value of Riskscore. Using the “timeROC” R package, the Riskscore showed an AUC of 0.61, 0.68, 0.7, 0.77, and 0.69 for 1-, 2-, 3-, 4-, and 5-year prognostic evaluation in the TCGA-LUAD training cohort, which validated its robustness in the OS evaluation (Figure 3c). Similarly, LUAD patients in the low-risk group demonstrated markedly better OS than in the high-risk group in both the TCGA-LUAD test cohort (TCGA-LUAD test cohort: *p* < 0.00061; TCGA cohort: *p* < 0.0001, Figure 3d,f). At each time point, the test set and TCGA cohort displayed a high AUC value (TCGA-LUAD test cohort: 0.72, 0.6, 0.56, 0.55, and 0.55; TCGA cohort: 0.67, 0.64, 0.63, 0.65, and 0.62, Figure 3e,g), indicating that the Riskscore had an accurate stratification. Similarly, the results of the validation analysis in the GSE31210 dataset were consistent with the above findings that high-risk LUAD patients had poor prognostic outcomes than low-risk LUAD patients (*p* = 4e − 04, Figure 3h). Specifically, the AUC value for 1-, 2-, 3-, 4-, and 5-year OS assessment reached 0.81, 0.73, 0.65, 0.64, and 0.68 in the GSE31210 dataset, respectively (Figure 3i).

### 3.4. Prognostic Modeling and Immune Characterization

**Figure 4:**
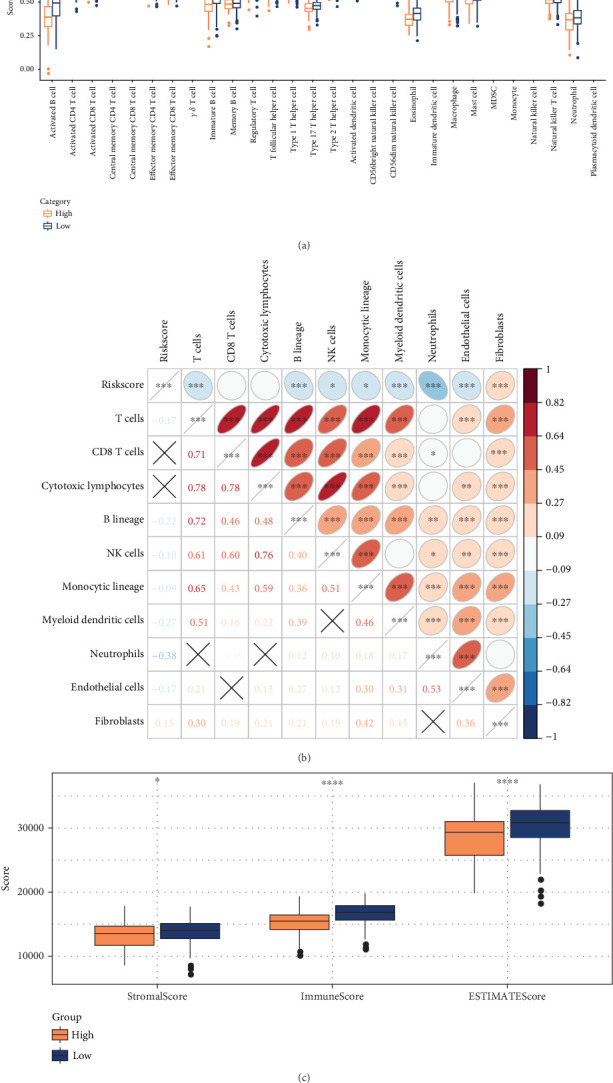
Immunologic characteristics between low- and high-risk subgroups of LUAD. (a) Immune infiltration in the two risk groups was analyzed by ssGSEA assessment. (b) Correlation between cellular infiltration score and the Riskscore for MCP. (c) Calculation of immune infiltration by ESTIMATE method between different risk subgroups. ⁣^∗∗∗∗^*p* < 0.0001, ⁣^∗∗∗^*p* < 0.001, ⁣^∗∗^*p* < 0.01, ⁣^∗^*p* < 0.05, and Ns represents *p* > 0.05.

Among the TCGA-LUAD patients, assessment of the infiltration of 28 types of immune cells revealed that in comparison to the low-risk group, most immune cells, such as activated CD8 T cells, NK cells, and activated B cells, were less infiltrated in the high-risk group (Figure 4). This suggested that the immune system in patients in the high-risk LUAD group was less active. The MCP evaluation of immune cell infiltration revealed a negative relation between the Riskscore and the infiltration of immune cells, including neutrophils, T cells, and B lineage (Figure 4). ESTIMATE assessment demonstrated markedly higher ESTIMATEScore, StromalScore, and ImmuneScore in the low-risk LUAD group in comparison to the high-risk LUAD group (*p* < 0.05), meaning that LUAD patients characterized by a high risk by the Riskscore had lower immune cell infiltration (Figure 4).

### 3.5. Riskscore in Relation to Gene Mutations and Oncogenic Pathways

**Figure 5:**
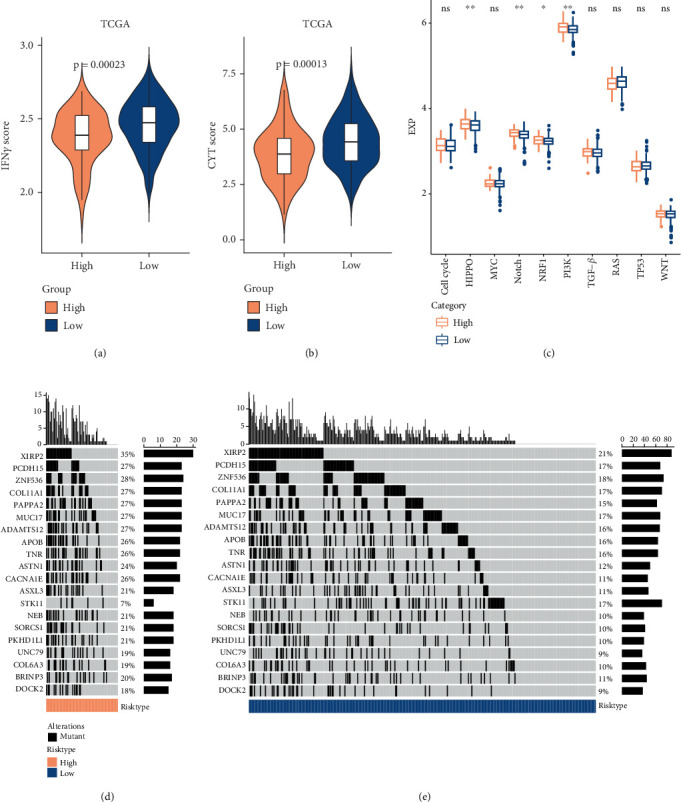
Analysis on cancer-related pathways and mutated genes in LUAD in the TCGA cohort. (a) IFN-*γ* score distribution difference. (b) CTY score distribution difference. (c) Oncogenic pathway score distribution difference. (d) Analysis of somatic mutations in the high-risk LUAD group. (e) Somatic mutation analysis in the low-risk LUAD group. ⁣^∗∗^*p* < 0.01, ⁣^∗^*p* < 0.05, and Ns represents *p* > 0.05.

Studies found that CD8^+^ T lymphocytes in the TME generate IFN-*γ*, which could upregulate the expressions of IDO1 and PD-1/PD-L1 [42, 43]. IDO1 expression upregulation is positively related to both tumor cell metastasis and a poor prognosis for tumor patients [44, 45]. Here, it was observed that the IFN-*γ* score was considerably higher in the low-risk LUAD group (*p* = 0.00023), indicating that LUAD patients characterized by the Riskscore as having a low risk were more susceptible to metastases (Figure 5a). In the TCGA-LUAD cohort, the low-risk LUAD group showed markedly higher CYT scores than the high-risk LUAD group (*p* = 0.00013) (Figure 5b). Comparison of the oncogenic pathways between the two risk groups revealed that high-risk patients had notably higher scores for HIPPO, Notch, NRF1, and PI3K pathways (Figure 5c). Gene mutation analysis for the TCGA-LUAD cohort indicated that among the top 20 mutant genes in LUAD patients, high-risk LUAD patients had higher mutation rates in comparison to low-risk LUAD patients. For instance, *XIRP2*, *PCDH15*, and *ZNF536* had a frequency of 35%, 27%, and 28% in the high-risk group, respectively (Figure 5d), and dropped to 21%, 17%, and 18% in the low-risk group, respectively (Figure 5e).

### 3.6. Functional Enrichment Analysis for the Two Risk Groups

**Figure 6:**
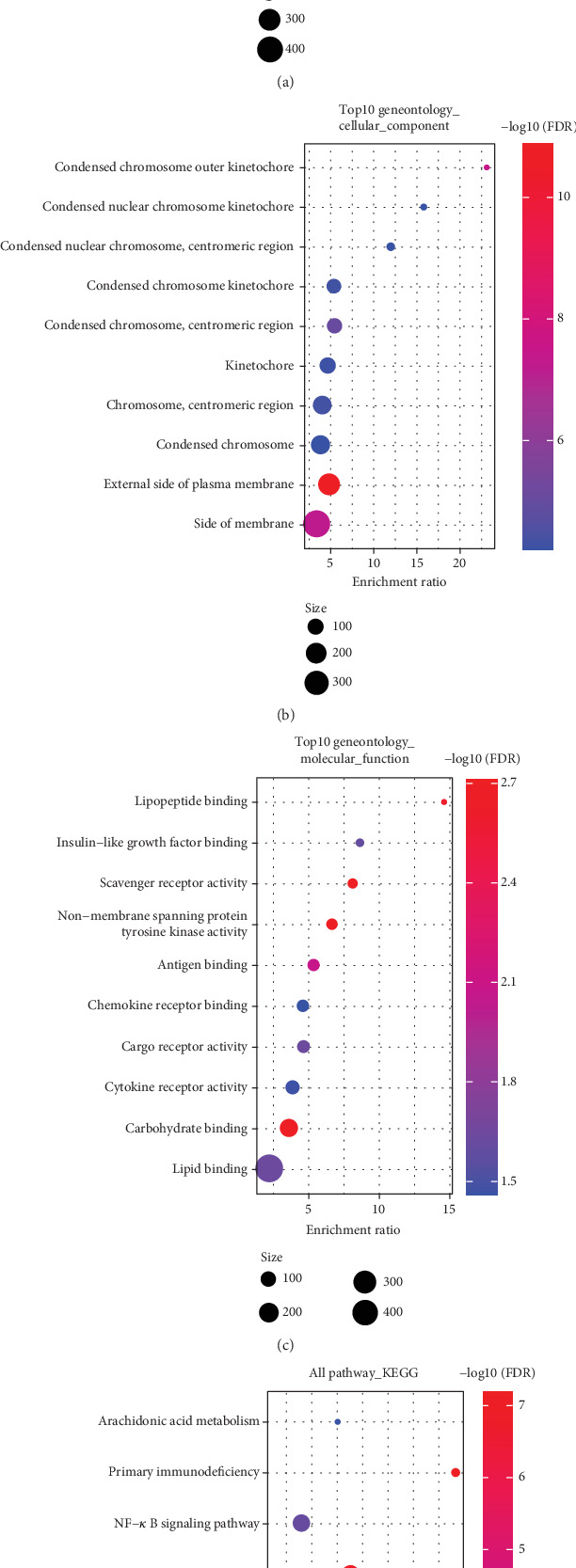
Functional enrichment analysis of the DEGs in low- and high-risk groups in TCGA-LUAD dataset. (a) The enriched DEGs in BP term. (b) The enriched DEGs in CC term. (c) The enriched DEGs in MF term. (d) KEGG annotations of the DEGs.

In the TCGA-LUAD dataset, the DEGs (|FC| > 1.5 and FDR < 0.05) between the two risk groups were selected using “limma.” Finally, we obtained 568 DEGs in the high-risk group, including 395 downregulated genes and 173 upregulated genes. Then, KEGG and GO analyses were performed on the DEGs. Specifically, the DEGs were mainly involved in adaptive immune response and some other functions in the GO-BP term (Figure 6a). In the GO-CC term, the DEGs were mainly localized in components such as the external side of the plasma membrane (Figure 6b). In the GO-MF term, the DEGs were mainly implicated in functions such as carbohydrate binding (Figure 6c). KEGG analysis revealed that these DEGs were markedly enriched in pathways such as hematopoietic cell lineage (Figure 6d). These findings demonstrated that these DEGs were significantly enriched in pathways correlated with metabolism.

### 3.7. Immunological Characterization of Patients in High- and Low-Risk Groups

**Figure 7:**
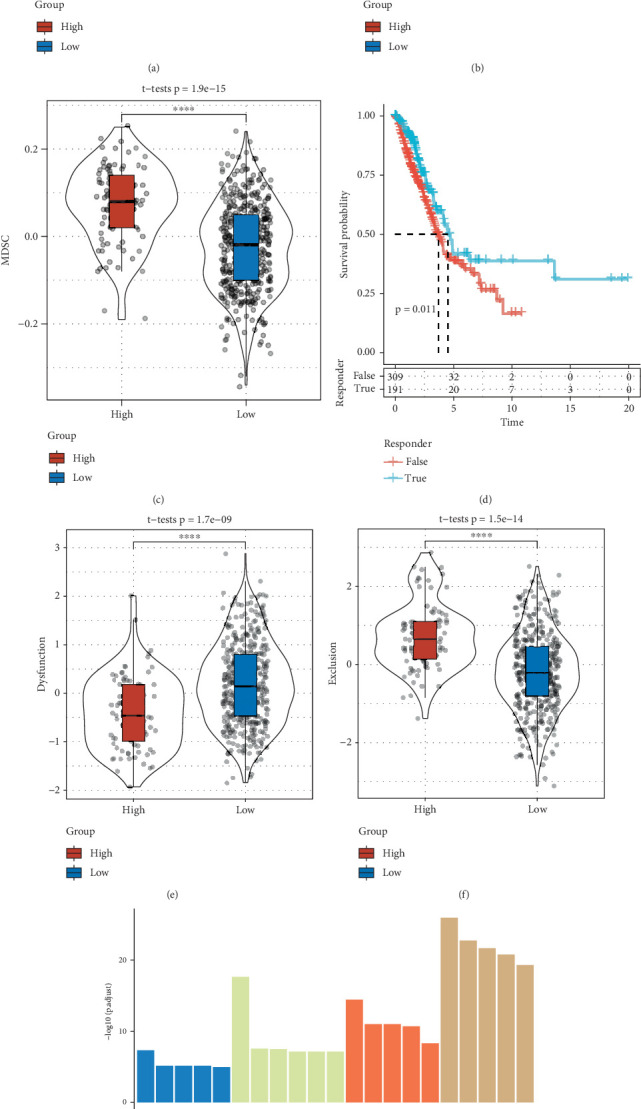
Analysis of the immunotherapy characteristics of patients in low- and high-LUAD subgroups. (a) TIDE score differences across risk subgroups of TCGA-LUAD. (b) CAF score differences between the two risk groups. (c) MDSC score differences between the two groups of TCGA-LUAD. (d) Survival difference between TCGA-LUAD immunotherapy subgroups. (e) Dysfunction score differences between the two groups of TCGA-LUAD. (f) Exclusion score differences between the two groups of TCGA-LUAD. (g) Functional annotation analysis of lncRNA-associated mRNAs. ⁣^∗^*p* < 0.05 and ⁣^∗∗∗∗^*p* < 0.0001.

The high-risk group in the TCGA-LUAD cohort displayed enhanced immunosuppressive characteristics, with significantly increased TIDE, cancer-associated fibroblast (CAF), and myeloid-derived suppressor cell (MDSC) scores relative to the low-risk group (Figures 7, 7, and 7). This indicated greater immunotherapy benefits for the high-risk patients who were more prone to have immunological escape. According to our analysis of the survival and immunotherapy response status predicted by the TIDE software, the survival of the true subgroup, which responded to immunotherapy, was considerably better than the false subgroup (*p* = 0.011, Figure 7). Additionally, relative to low-risk LUAD patients, high-risk LUAD patients had lower levels of dysfunction and higher levels of exclusion (Figure 7). Finally, to assess the role of the three prognostic lncRNAs, the mRNAs (Cor > 0.25 and *p* < 0.05) positively correlated with the lncRNAs were identified according to the Pearson correlation between the mRNAs and lncRNAs in the TCGA dataset. The mRNAs were then subjected to KEGG and GO enrichment analysis using the R package “WebGestaltR.” We found that the mRNAs linked to the three key lncRNAs were mainly involved in immune-correlated pathways, including T-cell activation and leukocyte differentiation (Figure 7).

### 3.8. Potential Effects of LINC01537 on the Migration and Invasion of LUAD Cells

**Figure 8:**
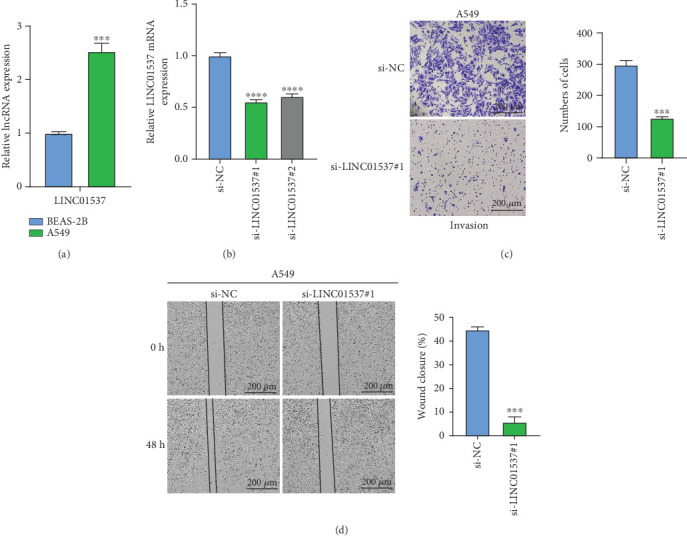
Examining the roles of LINC01537 in LUAD. (a) LINC01537 expression in A549 and BEAS-2B cells was detected by qRT-PCR. (b) Validation of knockdown efficiency of LINC01537 in A549 cells. (c) Representative photographs and the statistical analysis for the invasive cell counts in A549 cells after the knockdown of LINC01537 in the transwell assay. (d) Representative photographs and the statistical analysis for A549 cells after the knockdown of LINC01537 in the wound healing assay. SD ± mean was used to express the data: ⁣^∗∗∗^*p* < 0.001 and ⁣^∗∗∗∗^*p* < 0.0001.

Here, the expressions and functions of key genes in LUAD were examined. It should be noted that the relatively recent discovery of AC023421.1 and AL034397.3 genes and the unavailability of their gene sequences limited our analysis. Therefore, the present functional studies focused exclusively on the LINC01537 gene. First, the result of qRT-PCR showed that LINC01537 was notably upregulated in A549 cells than in BEAS-2B cells (Figure 8a, *p* < 0.001). LINC01537 has been reported to enhance the proliferation, invasion, and migration of gastric cancer cells [46]. Therefore, we explored the potential biological functions of LINC01537 by knockdown in A549 cells (Figure 8b, *p* < 0.001). Here, we examined the effects of the LINC01537 gene on the invasion and metastasis of A549 cells by wound healing assay and transwell assay. It was observed that knockdown of LINC01537 significantly inhibited the migration (*p* < 0.001) and metastasis (*p* < 0.01) of A5H49 cells (Figure 8c,d).

## 4. Discussion

Research showed that starvation-stimulated cancer cells promote tumor cell growth by inducing autophagy and glycolytic metabolism [47]. Ren et al. discovered that fasting therapy stimulates an antitumor immune response, increases immunogenic cell death, and efficiently kills tumor cells [48]. However, investigations into SRRGs in LUAD remain limited. To bridge the gap in research, this study examined the interaction between the SRRGs and LUAD. We developed a new system applying computational and statistical techniques for risk assessment in LUAD by integrating both glycolysis-associated and autophagy-associated SRRGs. The ROC curve analysis demonstrated that the Riskscore developed using three pivotal genes had a high efficacy for OS assessment among LUAD patients.

The present Riskscore consisted of three lncRNAs linked to the prognosis of LUAD, namely, *AC023421.1*, *AL034397.3*, and *LINC01537*. *AL034397.3* and *LINC01537* are associated with LUAD development. Guo et al. created a predictive risk model containing *AL034397.3* to predict the immunological state, immunotherapy response, chemotherapy, targeted treatments, and prognosis for high- and low-risk LUAD patients [49, 50]. *LINC01537* prevents tumor cell growth and metastasis through altering energy metabolism including the glycolytic metabolism of tumor cells to promote susceptibility to chemotherapy, showing a favorable correlation with the OS in lung cancer [51]. These results suggested that the key genes linked to starvation, autophagy, and glycolysis in LUAD may have an impact on tumor cell proliferation, metastasis, immune response, and drug resistance. At present, studies on lncRNA *AC023421*.*1* are limited. Therefore, further experiments exploring their roles in LUAD and the underlying molecular mechanisms are required in order to provide a better understanding for LUAD treatment.

By delivering alarm signals, the adaptive immune response modifies the tumor immunological microenvironment to promote the efficacy of immunotherapies [52]. The anticancer efficacy of anti-PD-1 immunotherapy resistance is restored when the adaptive immune response is activated in LUAD [53]. Recent studies reported that translocation of multifunctional proteins expressed on the surface of cancer cells outside the plasma membrane could promote tumor formation, inflammation, adhesion, leukocyte trafficking, and cell differentiation [54, 55]. It has been found that the outer plasma membrane pathway in LUAD is enriched with genes linked to a poor prognosis and their closely interacting immune genes, collectively contributing to tumor progression [56]. Furthermore, some carbohydrate chains present in tumor cells during carcinogenesis bind to leukocyte immunological receptors to suppress the anticancer immune response, showing the potential to serve as a novel target for cancer immunotherapy [57]. A previous study reported that aggressive LUAD is related to the loss of some hematopoietic cells [58]. Here, we also discovered that the DEGs between the two risk groups of LUAD were primarily enriched in the pathways of hematopoietic cell lineage, external side of the plasma membrane, carbohydrate binding, and adaptive immune response.

Genetic amplifications, deletions, and gene fusions in HIPPO can lead to tumor therapy resistance and tumor immunogenicity [59]. The transduction of the HIPPO signaling pathway promotes CAF recruitment and LUAD progression within the TME [60]. Notch signaling is crucial for controlling cell fate, tissue homeostasis, and organ development in postnatal animals. Dysregulated Notch signaling pathway enhances angiogenesis and epithelial–mesenchymal transition (EMT) in malignant tumors, which in turn contributes to tumor invasion, metastasis, and proliferation [61]. Upregulated NRF1 expression in colorectal cancer activates mitochondrial metabolism and biosynthesis, promoting the growth, invasion, and metastasis of tumor cells [62]. Tumor cell metastasis and partial angiogenesis can be elevated by the Notch axis-dependent regulation in LUAD [63]. In LUAD, brain metastasis is promoted when the PI3K pathway is activated [64]. The present study discovered that high-risk LUAD patients with a worse prognosis had significantly higher pathway scores for HIPPO, Notch, NRF1, and PI3K. It has been reported that highly modified tumors contain a higher burden of neoantigens, which could cause immunogenicity. In hepatocellular carcinoma, *XIRP2* mutations are closely linked to a poor prognosis and reduced chemosensitivity [65]. Furthermore, metastatic adnexal sebaceous gland cancer of the eye is discovered to have a substantial mutation in *PCDH15* [66]. *ZNF536* mutation is linked to a longer survival in LUAD but a worse prognosis in small-cell lung cancer and metastatic LUAD [67]. Notably, we found that *XIRP2* (35%), *PCDH15* (27%), and *ZNF536* (28%) had high mutation frequencies in the top 20 mutated genes in LUAD, indicating that high mutation frequencies of these genes may contribute to a worse prognostic outcomes in LUAD.

Immune cell infiltration is regarded as a crucial indicator of the tumor immune microenvironment. To effectively control cancer progression, infiltrating T cells should persist in the tumor site, migrate among tumor cells, and interact with the tumor cells [68]. Cancer patients with extensive immune cell infiltration between tumor cells have a more favorable prognostic outcome than those with infiltration limited to the perivascular region [69]. Intralesional immune cells are related to a better prognosis for patients with cancers such as colorectal and breast cancers [70, 71]. The present immune microenvironment analysis employing the Riskscore demonstrated that the infiltrating abundance of the majority types of immune cells, including NK cells, activated CD8 T cells, and activated B cells, was significantly increased in the low-risk group with a better prognosis. It has been reported that MDSCs directly promote the growth and spread of tumors. T-cell migration and proliferation are inhibited, T-cell and NK-cell death is triggered, immune effector cell function is suppressed, and antitumor reactivity mediated by T cells is inhibited by MDSC through its interaction with the PD-1 receptor [72]. CAF controls the biology of cancer cells and some other stromal cells in the TMEs through releasing various regulatory factors, synthesizing and altering extracellular mesenchyme, and promoting tumor recurrence and treatment resistance [73]. We observed that high-risk LUAD patients with a poor prognosis had notably higher infiltration of MDSCs and CAFs in comparison to the low-risk group. Patients with a worse prognostic outcome had a higher TIDE score, suggesting a lower rate of ICI therapy response. Overall, infiltration of MDSCs and CAFs and less active response to ICI therapy may result in the worse prognosis in LUAD.

There were certain limitations in the present work. Firstly, the size of samples from a public database for analysis was small. Secondly, prospective studies are required to validate the potential bias caused by the retrospective recruitment of patients. Furthermore, there was a lack of experimental and clinical validation for the biological activities of the key genes in our model. Hence, multicenter randomized controlled studies with higher quality, larger sample sizes, and sufficient follow-up data are required for further confirmation.

## 5. Conclusion

The present research showed that starvation-induced autophagy and glycolytic metabolism played important roles in the pathogenesis of LUAD. Employing multiple computational methods, we identified lncRNAs *AC023421.1*, *AL034397*.*3*, and *LINC01537* as the potential biomarkers for LUAD. Specifically, the three markers might affect LUAD progression by influencing gene mutations and immune infiltration in the tumor. Further validation of these genes and their potential regulatory drugs will improve our understanding of the roles of starvation-induced autophagy and glycolytic metabolism in the immune milieu of LUAD, contributing to the development of personalized treatment strategies and targeted immunotherapies.

## Figures and Tables

**Table 1:** The primer sequences for qRT-qPCR used in the study.

**Gene name**	**Forward primer**	**Revers primer**
LINC01537	5⁣′TTGTGCAGAGGAAGCTCTCAG 3⁣′	5⁣′TCCGCCTTTGTTTTCCTTCC 3⁣′
GAPDH	5⁣′ ACAACTTTGGTATCGTGGAAGG 3⁣′	5⁣′ GCCATCACGCCACAGTTTC 3⁣′

## Data Availability

The public dataset used in this study is available in GSE31210 (https://www.ncbi.nlm.nih.gov/geo/query/acc.cgi?acc=GSE31210).

## Nomenclature

LUAD: lung adenocarcinoma
WGCNA: weighted gene coexpression network analysis
OS: overall survival
TME: tumor microenvironment
SRRGs: starvation response-related genes
GRGs: glycolysis-related genes
ARGs: autophagy-related genes
lncRNA: long noncoding RNA
TCGA: The Cancer Genome Atlas
GEO: Gene Expression Omnibus
HADb: Human Autophagy Database
ESTIMATE: Estimation of STromal and Immune cells in MAlignant Tumor tissues using Expression data
DSigDB: Drug Signatures Database
TIDE: tumor immune dysfunction and rejection
ICI: immune checkpoint inhibition
DEGs: differentially expressed genes
CC: cellular component
MF: molecular function
BP: biological process
GO: Gene Ontology
KEGG: Kyoto Encyclopedia of Genes and Genomes
GSVA: gene set variant analysis
ssGSEA: single-sample gene set enrichment analysis
